# Harnessing endogenous pathways and metabolites to treat or prevent neurodegenerative disease

**DOI:** 10.1186/1750-1326-8-S1-O36

**Published:** 2013-09-13

**Authors:** Todd E Golde, Kevin Felsenstein, Paramita Chakrabarty, Andy Li, Brenda Moore, Jung Joo In, Pedor Cruz, Ashely Price, David Borchelt, Christopher Janus, Carolina Cebballos-Diaz, Awilda Rosario

**Affiliations:** 1 Center for Translational Research in Neurodegenerative Disease, Department of Neuroscience, McKnight Brain Institute, College of Medicine, University of Florida, Gainesville, FL, USA

## 

As the field moves towards earlier interventions in preclinical stages of AD or even envisions true primary prevention strategies, the therapeutic strategies employed must be “safe enough”. Whether any current therapeutic (e.g., β-secretase inhibitors, γ-secretase modulators (GSM) or anti-Aβ immunotherapies) being tested for disease modification is sufficiently safe is not known. In order to try and identify “safe enough” therapeutics, we have been evaluating whether endogenous regulators of Aβ can be harnessed as therapeutics. We have identified a cholesterol metabolite, cholestenoic acid (CA), as potent GSM, and have genetic data from mice that are consistent with a role for CA in regulating Aβ42 levels in the brain. We will discuss our ongoing studies exploring how we might utilize CA or the CA metabolic pathway to safely lower Aβ42 levels. In addition, we have been evaluating how we can use soluble forms of endogenous innate immune receptors to alter Aβ deposition and modulate neuroinflammation in mouse models of AD. We have also been exploring the utility of such strategy in other neurodegenerative models. Effects of soluble toll-like receptors and soluble TREM2 will be presented. Our current data establish that soluble TLR4 and 5 both dramatically inhibit Aβ deposition but that soluble TLR2 and 6 do not. We also have data that suggest the utility of these soluble receptors in α-synucleinopathies. By using endogenous metabolites and receptors, we hope that the likelihood of adverse toxicities will be diminished and that these or endogenous factors can be safely developed for prophylactic or early intervention in AD and perhaps other neurodegenerative disorders.

